# Vitamin D3 status and seroprevalence of *Toxoplasma gondii* in reproductive-aged women in Northern Iran

**DOI:** 10.1186/s12905-025-04114-2

**Published:** 2025-11-06

**Authors:** Mostafa Tork, Mitra Sadeghi, Shahabeddin Sarvi, Fatemeh Khazaei, Bahareh Basirpour, Somayeh Ahmadi, Seyyed Ali Shariatzadeh, Seyed Abdollah Hosseini, Marzieh Zamaniyan, Ahmad Daryani, Sargis A. Aghayan

**Affiliations:** 1 Toxoplasmosis Research Center, Communicable Diseases Institute, Mazandaran University of Medical Sciences, Sari, Iran; 2 Student Research Committee, School of Medicine, Mazandaran University of Medical Sciences, Sari, Iran; 3 Department of Parasitology and Mycology, Faculty of Medicine, Mazandaran University of Medical Sciences, Sari, Iran; 4 Department of Medical Sciences, Sha. C., Islamic Azad University, Shahrood, Iran; 5 Department of Medical Sciences, Biological Nanoparticles in Medicine Research Center, Shahrood Branch, Islamic Azad University, Shahrood, Iran; 6 Diabetes Research Center, Mazandaran University of Medical Sciences, Sari, Iran; 7 Sexual and Reproductive Health Research Center, Mazandaran University of Medical Sciences, Sari, Iran; 8 Laboratory of Molecular Parasitology, Scientific Center of Zoology and Hydroecology, NASRA, 7p. Sevak St, Yerevan, Armenia; 9 Laboratory of Zoology, Research Institute of Biology, Yerevan State University, 1 Alex Manoogian, Yerevan, 0025 Republic of Armenia

**Keywords:** Zoonotic diseases, Vitamin D3 deficiency, Women of reproductive age, Prevalence, Serological testing, Northern Iran

## Abstract

**Background:**

*Toxoplasma gondii* (*T. gondii)* seropositive is a significant public health concern due to its potential impacts on both general morbidity and specific risks to maternal-fetal health. Concurrently, insufficient Vitamin D3 (Vit D3) levels during gestation may contribute to adverse outcomes for both mother and developing fetus. This study was designed to examine the Vit D3 status and seroprevalence of *T. gondii* among women of reproductive age in northern Iran.

**Methods:**

This investigation employed Enzyme-Linked Immunosorbent Assay (ELISA) to quantify anti-*T. gondii* immunoglobulins (IgG/IgM) and Vit D3 levels in 320 serum specimens from women of reproductive age in northern Iran. Data analysis incorporated prevalence calculations, binary, and multivariate logistic regression for odds ratio determination using SPSS v21.

**Results:**

A total of 245 out of 320 (76.56%) women of reproductive age were characterized as Vit D3 deficient. Serological analysis revealed a 61.9% (198/320) IgG seroprevalence rate for *T. gondii*, with only 0.3% (1/320) showing IgM positivity. Notably, 159 seropositive cases (64.8%) occurred among Vit D3-deficient participants. Statistical evaluation demonstrated a significant correlation (OR = 1.71; 95% CI: 1.01–2.88; *P* = 0.045), indicating 71% increased *T. gondii* seropositive risk in individuals with insufficient Vit D3 levels compared to those with adequate status.

**Conclusions:**

This study reveals a high concurrent prevalence of *T. gondii* infection and Vit D3 deficiency among women of reproductive age in northern Iran. Vit D3 deficiency may be linked to higher susceptibility to infection and potentially to reproductive health issues. However, due to the cross-sectional design of the study, it is not possible to determine whether Vit D3 deficiency increases the risk of infection or if infection itself influences Vit D3 levels. These findings highlight a significant association that warrants further prospective and interventional studies to clarify the direction and underlying mechanisms of this relationship.

**Supplementary Information:**

The online version contains supplementary material available at 10.1186/s12905-025-04114-2.

## Background

*Toxoplasma gondii* (*T. gondii*) is an obligate intracellular coccidian protozoan responsible for causing *T. gondii* seropositive. In humans, infection usually occurs through ingestion of contaminated water or food, or consumption of raw or undercooked meat from infected animals such as sheep, goats, pigs, and cattle, which can serve as intermediate hosts of the parasite [1]. Transmission may also occur through less frequent pathways such as vertical transmission, transfusion of infected blood, or transplantation of contaminated organs, as documented in previous studies [2–4].

Research in Iran shows highly variable *T. gondii* prevalence, ranging from 6% in healthy schoolchildren to 60% in pregnant women [5]. The higher rates and detection of recent infections in high-risk groups, such as pregnant women with diabetes, highlight significant regional differences and underscore the infection’s particular clinical importance for vulnerable populations [6–8].

*T. gondii* seropositive is linked to distinct neurological and neuropsychiatric symptoms [9]. Research has shown that individuals infected with *T. gondii* tend to have an increased incidence of depression [10], and heightened anxiety levels [11]. The primary concern of *T. gondii* seropositive in pregnant women is the transmission of the parasite to the fetus [12]. This can lead to serious complications, including miscarriage, premature delivery, intracerebral calcifications, retinopathy, neuropathy, progressive hydrocephalus, microcephaly [13, 14]. Several studies have identified a link between *T. gondii* seropositive and infertility [15, 16].

Vitamin D3 is a steroid hormone and plays a crucial role in maintaining a healthy immune system [17]. The presence of Vit D3 receptors on peripheral blood monocytes and activated lymphocytes in humans indicates that Vit D3 acts as an immunomodulatory agent. Also, Vit D3 deficiency has been linked to various fertility issues [18]. Supplementation and maintaining optimal Vit D3 levels have been shown to improve fertility indicators reduce pregnancy-related risks [19, 20].

The overlap between the spectrum of neuropsychiatric disorders and chronic physical illnesses linked to both *T. gondii* infection and Vit D3 insufficiency [21–23], as well as both in vivo and in vitro effects of Vit D3 on the proliferation of *T. gondii* [24], suggests that *T. gondii* seropositive may be associated with lower Vit D3 levels, which could contribute to the observed connection between *T. gondii* seropositive and the various diseases and disorders mentioned above *T. gondii* infection may be associated with lower Vit D3 levels, potentially affecting the host’s immune defenses; however, further studies are needed to confirm this relationship. As a result, this reduction in Vit D3 could contribute to a higher occurrence of various physical and neuropsychiatric conditions. It is also possible that individuals who already have lower Vit D3 levels are more prone to developing susceptible to *T. gondii* infection. This cross-sectional study aimed to investigate the Vit D3 status and *T. gondii* seroprevalence among women of reproductive age in northern Iran, with the goal of providing evidence to inform targeted public health measures and identify potential risk factors in this population.

## Materials and methods

### Ethical approval

The study protocol was approved by the Institutional Ethics Committee of Mazandaran University of Medical Sciences (Approval No. IR.MAZUMS.REC.1402.061) written informed consent was obtained from all participants before sample collection. The study adhered to the ethical principles outlined in the Declaration of Helsinki (2013 revision). To ensure participant confidentiality and privacy, all collected data were anonymized and securely stored.

### Study population

This cross-sectional study was conducted on a total of 320 serum samples from women of reproductive age were collected (consecutive sampling) from January 1, 2023, to March 30, 2024. The inclusion criteria for participation were as follows: females aged 18 to 40 years, residing in Mazandaran province (with average annual temperatures ranging from 16 to 18 °C and humidity around 70%), and able to provide informed consent. Individuals with a history of autoimmune diseases or currently undergoing immunosuppressive treatments, and female outside the 18–40 year age range were excluded from the study.

A structured questionnaire was completed for each participant, collecting information on age, education, place of residence (urban or rural), meat consumption (raw or undercooked), history of cat exposure, consumption of undercooked eggs, vegetable consumption, and contact with soil and body mass index (BMI).

A structured questionnaire was used to collect demographic data and information on potential risk factors. The questionnaire was designed based on previous studies and reviewed by experts to ensure content validity. It was primarily self-administered; however, for participants with low literacy, trained interviewers assisted in completing the questionnaire.

### ELISA procedure

Sera were tested in duplicate for anti-*T. gondii* IgG and IgM using an ELISA kit (Pishtaz Teb, Iran; 100% Sensitivity, 99% specificity for anti-*Toxoplasma* IgM detection, 100% sensitivity and 100% specificity for anti-*Toxoplasma* IgG detection) following the manufacturer’s instructions. In cases of discrepancies between duplicate measurements, the mean optical density was calculated. Serum Vit D3 levels were measured using commercial kits, expressed in ng/mL, and categorized based on the reference ranges provided by the kit: <30 ng/mL as insufficient and ≥ 30 ng/mL as sufficient.

### Statistical analysis

Data from the ELISA tests and questionnaire were analyzed using SPSS version 21. Binary logistic regression was used to estimate odds ratios (OR) with 95% confidence intervals (CI). Multivariate logistic regression models were adjusted for potential confounders such as age, cat contact, and undercooked meat consumption. A 95% confidence interval was used, and p-values less than 0.05 were considered statistically significant.

## Results

A total of 245 out of 320 (76.56%) women of reproductive age were characterized as Vit D3 deficient and only 75 cases (23.44%) had sufficient Vit D3. Among 320 samples, 198 samples (61.88%) for IgG and 1 sample (0.31%) for IgM antibodies against *T. gondii* were positive.

The results showed (Table 1) that the prevalence of IgG antibodies against *T. gondii* increased with age, ranging from 50% in participants under 25 to 69.1% in participants over 35 years of age. The data analysis also showed a significant relationship between the prevalence of *T. gondii* and the age in the women under study (*P* = 0.037).

**Table 1:** Seroprevalence of *Toxoplasma gondii* in women of reproductive age in Mazandaran province, Iran by different variables

Variables		No.	Positive [%]	Odds ratio [95%CI]	*P*- value
Age	< 25	12	6 (50%)	1	
	25–30	119	63 (52.9%)	1.12 (0.42–3.88)	0.85
	30–35	108	73 (67.5%)	2.08 (0.59–7.28)	0.25
	> 35	81	56 (69.1%)	2.74 (1.01–6.52)	0.04
Residence	Urban	236	141 (59.7%)	1.47 (0.84–2.50)	0.15
	Rural	84	57 (67.8%)		
Education	None-Academic Educated	117	77 (65.8%)	1.30 (0.81–2.10)	0.27
	Academic Educated	203	121 (59.6%)		
Cat exposure history	Yes	98	71 (72.4%)	1.97 (1.18–3.28)	0.009
	No	222	127 (57.2%)		
Soil exposure history	Yes	103	65 (63.1%)	1.08 (0.67–1.76)	0.76
	No	217	133 (61.2%)		
Eggs consumption	Raw/Half Cooked	111	69 (62.1%)	1.02 (0.63–1.64)	0.94
	Fried/Cooked	209	129 (61.7%)		
Consume meat	Cooked	156	109 (69.8%)	1.95 (1.23–3.12)	0.004
	Half Cooked	164	89 (54.2%)		
Vegetable’s consumption	Raw/unwashed	213	139 (65.2%)	1.65 (0.98–2.64)	0.06
	Cooked	107	59 (55.1%)		
BMI	< 25	15	8 (53.33%)	1	
	25.1–30	146	89 (60.96%)	1.36 (0.45–4.08)	0.57
	30.1–35	118	78 (66.11%)	1.71 (0.55–5.17)	0.35
	> 35	41	23 (56.09%)	1.12 (0.33–3.77)	0.86
Vitamin D3	Deficiency	245	159 (64.8%)	1.71 (1.01–2.88)	0.045
	Sufficient (Healthy)	75	39 (52.0%)		
Total		320	198 (61.88%)		

A notable finding in this study was the statistically significant association between cat exposure history and IgG seropositivity (OR = 1.97; 95% CI: 1.18–3.28; *P* = 0.009). As the results of this study show, individuals who reported contact with cats had a higher seropositivity rate (72.4%) compared to those without such contact (57.2%).

Also, consumption of undercooked meat was significantly associated with higher seroprevalence (OR = 1.93; 95% CI: 1.21–3.10; *P* = 0.004). Other variables, including residence (*P* = 0.189), contact with soil (*P* = 0.755), raw egg consumption (*P* = 0.94), and type of vegetable consumption (*P* = 0.07), BMI > 35 (*P *= 0.86) and educational level (*P* = 0.271) did not show statistically significant associations with seroprevalence of *T. gondii.*

A total of 320 participants were enrolled in the study, of whom 75 individuals (23.4%) had sufficient Vit D3 levels, while 245 individuals (76.6%) had insufficient levels. Among those with insufficient Vit D3 (*n* = 245), 159 samples (64.8%) tested positive for IgG, and only one sample (0.4%) was positive for IgM specific to *T. gondii*. The true prevalence for *T. gondii* IgG in the Vit D3-insufficient group was estimated at 66.1% (95% CI: 59.9–71.9) and for IgM at 0.4% (95% CI: 0.01–2.2). In contrast, among those with sufficient Vit D3 levels (*n* = 75), 39 samples (52%) were IgG positive, and all tested negative for IgM antibodies against *T. gondii* (true prevalence IgG: 53.7%, 95% CI: 41.2–65.9; IgM: 0%, 95% CI: 0–4.8) (Table 1).

The overall mean serum Vit D3 level among the 320 participants was 17.5 ± 8.2 ng/mL, with a minimum of 7.0 ng/mL and a maximum of 43.0 ng/mL. Among participants, those with insufficient Vit D3 levels had a higher prevalence of *T. gondii* infection compared to those with sufficient levels. Statistical analysis showed that this corresponded to a 1.71-fold increased risk (OR = 1.71; 95% CI: 1.01–2.88; *P* = 0.045). Multivariable logistic regression further identified age > 35 years, Vit D3 deficiency, history of cat exposure, and consumption of undercooked meat as independent predictors of *T. gondii* seropositivity (Table 2).

**Table 2:** Multivariate logistic regression analyses of the predictors for toxoplasmosis among women of reproductive age in Northern Iran

Variable	Coefficient	Standard Error	*p*-value	Odds Ratio	95% Confidence Interval
Age > 35	0.93	0.33	0.004	2.53	(1.34–4.81)
Vit D3 deficiency	0.67	0.30	0.024	1.96	(1.09–3.52)
History of Cat exposure	1.01	0.30	0.001	2.73	(1.51–4.96)
Consumption of half cooked meat	0.64	0.25	0.011	1.91	(1.16–3.13)
Constant	−0.81	0.29	0.006		

## Discussion

The findings highlight a high seroprevalence of *T. gondii* and a potential link to Vit D3 deficiency among women of reproductive age, underscoring a major public health concern in northern Iran. Various studies, including those by Khademi et al.., (2019), Mousavi et al., (2020) in Iran, Rashed et al., (2021) in Saudi Arabia, and Jabber et al., (2021) in Iraq, have reported the prevalence of anti-*Toxoplasma* antibodies as 27%, 33.5%, 18.8%, and 42.1%, respectively [25–28]. The findings of this study show that the prevalence of *T. gondii* seropositive is high, which could be due to various reasons such as socio-cultural conditions, dietary habits such as eating undercooked meat, more contact with cats due to the abundance of cats in the region, and favorable weather conditions (with an average temperature of 16 to 18 degrees Celsius and humidity around 70%) for the survival of oocysts excreted by cats.

Age is one of the factors that can indirectly affect the prevalence of *T. gondii* seropositive in society by affecting individual and social behaviors. According to studies, increasing age is directly related to the chance of contracting *T. gondii* seropositive infection. The higher seroprevalence observed in older age groups may be related to longer cumulative exposure to risk factors, although the underlying mechanisms remain to be clarified [29].

In the present study, a significant relationship was observed between age and the seroprevalence of *T. gondii* infection. The results indicated that women under 25 years of age had the lowest prevalence, whereas the highest seroprevalence was observed among those aged 30–35 years. Similarly, Fan et al. (2007) [30] and Mostafavi et al. (2011) [31] reported that older individuals were more likely to be exposed to the infectious form of the parasite, with the greatest number of positive cases occurring in the 30–45 year age group.

Conversely, a study conducted in Croatia found that the highest incidence of *T. gondii* infection occurred in individuals younger than 15 years [32]. Such discrepancies in the peak ages of infection across different regions may be attributed to variations in climatic conditions, dietary patterns, and lifestyle practices that influence exposure at different stages of life. In Iran, the high prevalence of *T. gondii* infection among women of reproductive age is particularly concerning and highlights the urgent need for targeted preventive strategies for this vulnerable population.

Data from the present study suggest that undercooked meat consumption is significantly associated with higher exposure to *T. gondii*. Women who reported eating undercooked meat demonstrated a higher seroprevalence (69.87%) compared with those who consumed properly cooked meat (54.27%). Statistical analysis confirmed this association (*p* = 0.004), indicating that dietary habits play an important role in the transmission of toxoplasmosis.

These findings are consistent with several previous studies. Soltani et al., (2021) reported a significant correlation between raw or undercooked meat intake and *T. gondii* infection in pregnant women [33]. Similarly, Nurul Fadilah et al., (2020) observed that consuming undercooked meat increased the risk of infection by 9.7-fold in pregnant women [34]. Nematollahi et al., (2022) also highlighted meat consumption as a major transmission pathway, demonstrating a significant link between infection prevalence and meat preparation methods [35]. In contrast, Sarman et al., (2014) found no significant association, which was attributed to the relatively low prevalence of undercooked meat consumption in India [36].

The study demonstrated a difference of *T. gondii* seropositive in individuals with history of cat exposure (72.45%) versus without history of exposure (57.21%), with this difference being statistically significant (*p* < 0.01). These findings corroborate Ziad et al.‘s (2023) report of substantially higher infection rates among pet keepers (70% vs. 30%) [37]. Consistent with our results, Kanani et al., (2022) reported higher *T. gondii* antibodies prevalence in individuals exposed to cats versus unexposed controls [38].

This analysis revealed a significant association between Vit D3 deficiency and *T. gondii* infection in reproductive-aged women. The subjects with insufficient levels of Vit D3 presented significantly higher toxoplasmosis incidence (64.89%) and a 1.7-fold higher risk of infection (adjusted OR = 1.71; 95% CI: 1.01–2.88; *P* = 0.045). Importantly, this association remained significant after adjusting for major confounders including age, history of cat exposure, undercooked meat consumption, and educational level, supporting Vit D3 insufficiency as an independent risk factor for toxoplasmosis, supporting the possibility that Vit D3 status may influence host susceptibility.

These findings align with previous studies reporting a significant relationship between Vit D3 insufficiency and *T. gondii* seropositive. For instance, Huang et al., (2022) observed that 42.3% of *T. gondii* seropositive individuals had insufficient Vit D3 levels, significantly higher than seronegative individuals (*P* < 0.001), with an odds ratio of 1.303 [39]. Similarly, Tayeb et al., (2019) found a significantly higher seroprevalence of IgM antibodies against *T. gondii* (18.43%) among women with insufficient Vit D3 compared to controls (*P* < 0.05) [40]. Additionally, Rasheed et al. (2021) [28] and Kashan et al. (2019) [41] reported significant associations between *T. gondii* seropositive and Vit D3 deficiency (*P* < 0.001 and *P* < 0.05, respectively). However, contradicting these findings, al-Fakhar et al., (2022) found no statistically significant relationship between *T. gondii* seropositive and Vit D3 levels among 56 women with a history of miscarriage in Iraq (*P* = 0.386), differences in study design, population demographics, sample size, and seasonal or geographic variations in Vit D3 status may explain these discrepancies [42].

The strong link between Vit D3 deficiency and *T. gondii* infection is due to Vit D3’s role in regulating immune responses. Vitamin D3 modulates innate and adaptive immunity by inducing antimicrobial peptides like cathelicidins and enhancing Th1 responses, both of which are crucial for controlling intracellular pathogens such as *T. gondii* [43]. Deficiency may weaken immunity, increasing susceptibility to *T. gondii* seropositive. Studies show that patients with lower Vit D3 levels had more *T. gondii* infections.

This study has several limitations that should be considered when interpreting the findings. First, its cross-sectional design precludes any causal inference regarding the relationship between Vit D3 status and *T. gondii* infection. Second, lifestyle and dietary data were collected using self-reported questionnaires, which are inherently prone to recall bias. Third, several potential confounders that may influence both Vit D3 levels and susceptibility to infection (such as sunlight exposure, body mass index, overall diet quality, and socioeconomic status) were not measured or controlled for in this study. Finally, the sample was restricted to women residing in a single province of northern Iran, which may limit the generalizability of our results to broader populations.

## Conclusion

This study represents the first assessment of *T. gondii* seroprevalence in relation to Vit D3 status among women of reproductive age in northern Iran. Beyond a high prevalence of both Vit D3 deficiency and toxoplasmosis, our findings indicate a significant association between insufficient Vit D3 levels and higher *T. gondii* seroprevalence. However, the cross-sectional design precludes any inference of causality or temporal direction. The observed high prevalence of *T. gondii* seropositive underscores the need for targeted public health measures, including proper meat cooking, thorough washing of vegetables, and responsible pet care.

Although ensuring adequate Vit D3 through supplementation or lifestyle modifications may represent a potential preventive approach, the cross-sectional design of this study does not allow causal inferences. We recommend future prospective cohort studies to examine temporal associations, interventional trials to assess the protective role of Vit D3 supplementation, and studies in broader populations to generalize these findings. Such efforts could contribute to a clearer understanding of Vit D3’s potential role in host defense against *T. gondii* and inform evidence-based strategies to improve reproductive health outcomes.

## Supplementary Information

Supplementary Material 1

## Data Availability

The data that support the findings of this study are available from the corresponding author upon reasonable request.

## Abbreviations

Vit D3: Vitamin D3
*T. gondii*: *Toxoplasma gondii*
ELISA: Enzyme-linked Immunosorbent assay
BMI: Body mass index
