# Supplementary figures and images for: Vitamin D3 status and seroprevalence of Toxoplasma gondii in reproductive-aged women in Northern Iran
Source: BMC Womens Health. 2025 Nov 6;25:544. doi: 10.1186/s12905-025-04114-2 (PMC12593908; doi:10.1186/s12905-025-04114-2)

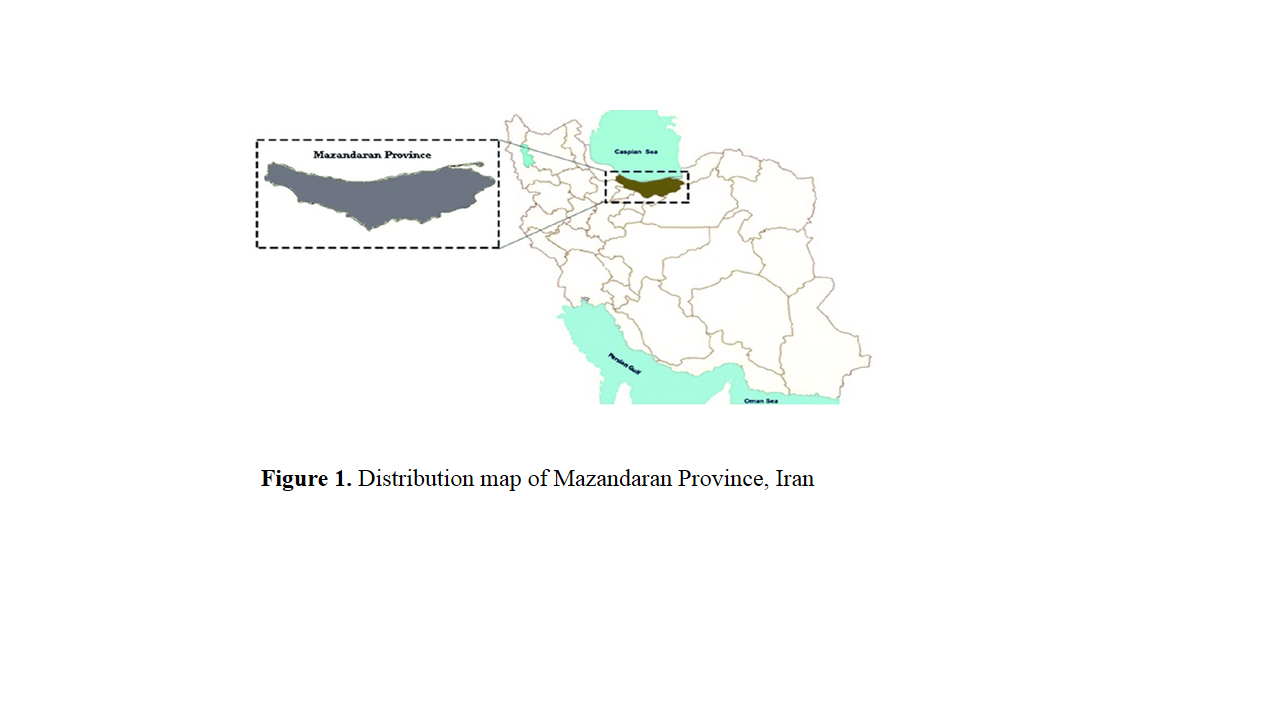

Supplement: Supplementary file 1 — Supplementary Material 1 [file 12905_2025_4114_MOESM1_ESM.tif]
